# A Pedunculated Eccrine Poroma Presented as a Skin Tag on the Right Thigh of a Female Individual: A Case Report

**DOI:** 10.7759/cureus.70964

**Published:** 2024-10-06

**Authors:** Abdulkarim Hasan, Khaled Ahmed Alsagheer, Mohamed Galal Abdelwahab Ali, Ahmed Mohammed Khatan, Mansour Hosny Ali Hassan, Abdallah Whaiba

**Affiliations:** 1 Department of Pathology, Faculty of Medicine, Al-Azhar University, Cairo, EGY; 2 Department of Laboratory, Al-Baha Health Cluster, Ministry of Health, Al-Baha, SAU; 3 Department of General Surgery, Al-Azhar University, Assiut Branch, Asyut, EGY; 4 Department of Surgery, Al Makhwah General Hospital, Al-Baha Health Cluster, Al Makhwah, SAU; 5 Department of General Surgery, General Organization for Teaching Hospitals and Institutes, Damanhour Teaching Hospital, Damanhour, EGY; 6 Department of Medicine, Broomfield Hospital, Mid and South Essex National Health Service (NHS) Foundation Trust, Essex, GBR

**Keywords:** eccrine adnexal tumor, eccrine poroma, excision surgery, histopathology examination, skin nodule

## Abstract

Poroma is a relatively rare benign adnexal neoplastic lesion that usually affects elderly patients in the extremities, with an unclear pathogenesis. It is notable for having a variety of morphological appearances that make diagnosis challenging, particularly when observed on an uncommon anatomical site. Here, we present a case of a woman in her mid-50s who presented with a skin pedunculated lesion over the right thigh. The lesion had a smooth surface and clinically mimicked a skin tag. The lesion was surgically excised and sent for histopathology. On microscopic examination, anastomosing cords and broad columns of more or less uniform basaloid cells extending to the dermis with no atypical changes were seen, confirming the diagnosis of eccrine poroma. It is emphasized that poromas should be considered in the clinical and histological differential diagnosis of nonacral skin lesions. Complete excision cures the condition and prevents local recurrence.

## Introduction

Poroma is a relatively rare benign adnexal neoplastic lesion that usually affects elderly patients. Its pathogenesis is unclear. However, recent genetic studies suggest gene fusions as the primary cause of oncogenetic pathways. Poroma usually presents as a solitary skin swelling, which can be skin-colored or pigmented and may be sessile or pedunculated. It may manifest as a papule or nodule, and is most commonly located on the soles or palms. In most cases, it is asymptomatic [1].

Eccrine and apocrine sweat gland neoplasms account for only 1% of all primary cutaneous lesions, but poromas are identified in around 10% of these lesions [2]. According to the literature, it arises from eccrine glands of the acral region, although it has been reported that it may have an apocrine origin. Approximately three million eccrine glands are distributed in the human body in many anatomical sites and different densities [3,4].

It is predominantly seen on the palms and soles. Thus, the appearance of eccrine poromas in other anatomical locations may not be expected [1]. However, less common locations have also been described, such as eyelids, buttocks, and the vulva [5-7]. The diagnosis of poroma of the thigh is exceedingly rare and uncommon, with only a few reported cases [8]. Here, we report a case of poroma with an unusual and infrequent location. However, it is a benign lesion and is completely excised with no local recurrence until the present.

## Case presentation

**Figure 1:**
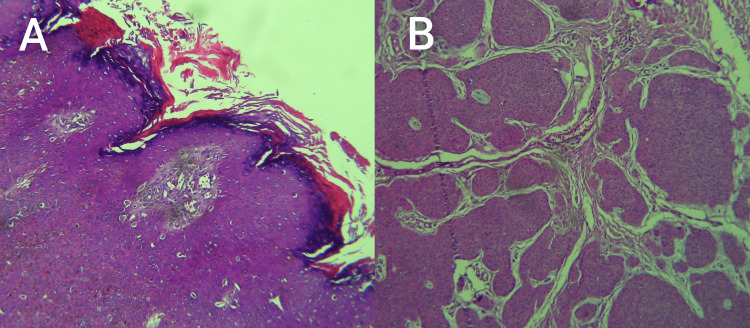
Histopathology of the epidermis and dermis (H&E, 100× original magnification). Histopathological picture of the lesion showing thick epidermis (A) with a neoplastic process exhibiting broad anastomosing bands demarcated from the stroma (B) H&E: hematoxylin and eosin

**Figure 2:**
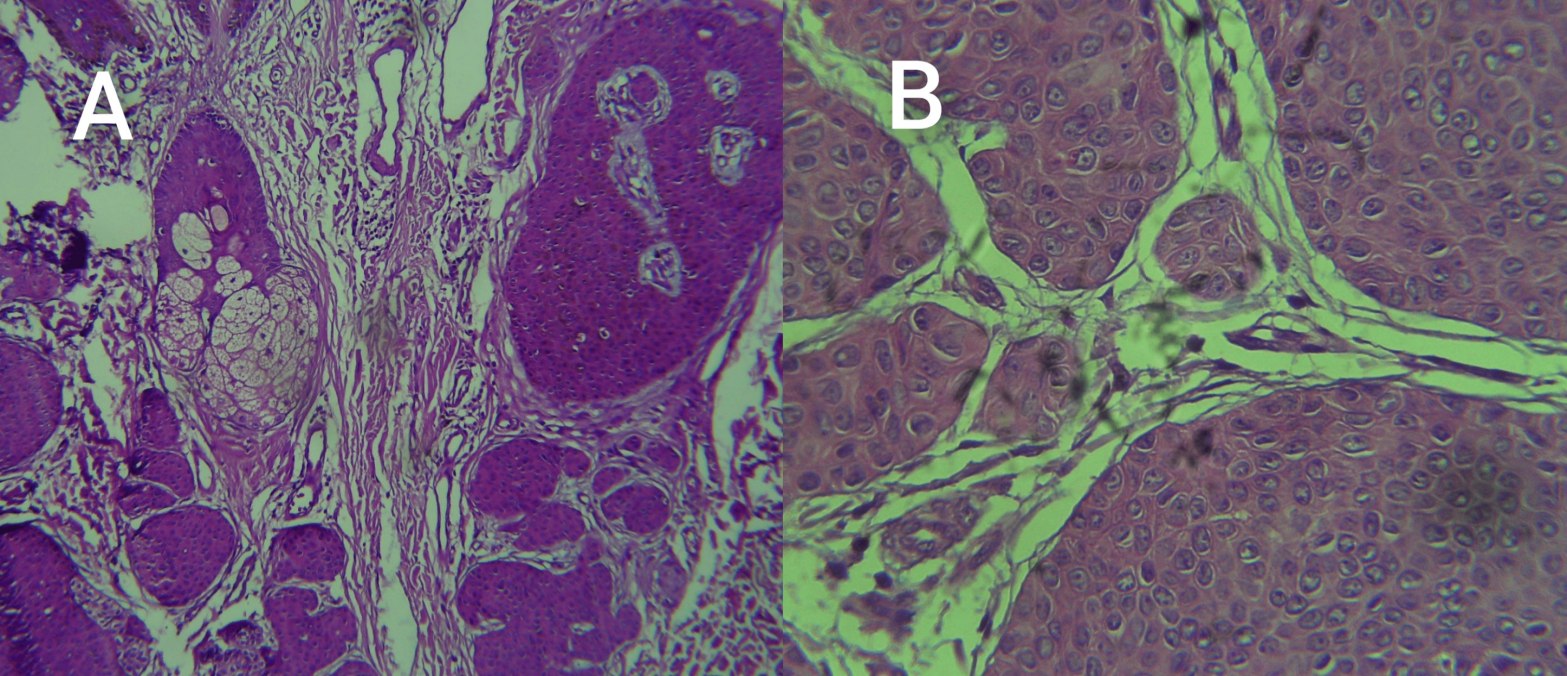
Histological features of the poroma with dermal extension (A) and a high-power picture showing the uniform basaloid cells (B) (H&E, 400×) H&E: hematoxylin and eosin

A 45-year-old woman with no previous related health problem presented to the surgery clinic with a skin polypoidal mass at the lateral side of her right thigh. Upon a physical examination of the right upper part of the thigh, a 1.5-cm protuberant, 1.3 cm in diameter, painless polypoidal soft tissue mass was observed. It had been discovered by the patient six weeks prior, had slowly increased in size, and occasionally itched. Because of this clinical appearance, a skin tag/fibroepithelial polyp was considered, and an excision biopsy was carried out as a safety margin. A gross examination in the histopathology laboratory revealed a polypoidal skin-covered mass measuring 1.5 × 1.4 cm with a soft grayish-white cut section located 0.1 cm from the least side margin. Histopathological examination of an epidermal covering with a neoplastic process exhibiting broad anastomosing bands demarcated from the stroma, composed of columns of uniform basaloid cells extending to the dermis and connected to the epidermis. Eosinophilic material was found filling certain cystic areas (Figures 1, 2).

The tumor was circumscribed well and presented with a rare inflammatory mononuclear cell infiltrate. Normal skin appendages were also observed in other regions. The absence of cytological atypia, increased mitotic activity, cellular pleomorphism, and necrotic changes supported the exclusion of the aggressive lesion, porocarcinoma, as a histologically challenging differential diagnosis. The patient was being followed up at the clinic for one year with no evidence of malignancy or local recurrence.

## Discussion

Cutaneous adnexal tumors include both benign and malignant neoplasms. Classification usually depends on the differentiation toward typical adnexal structures: follicular, eccrine, apocrine, and sebaceous. Adnexal tumors are relatively rare skin tumors, and clinicians find it difficult to diagnose and categorize them in most cases [9].

Eccrine and apocrine sweat gland adnexal tumors cover only around 1% of all primary skin lesions, and poromas account for approximately 10% of these lesions [1,2]. Poromas are often defined as great imitators since they share features of common benign and some malignant skin tumors in clinical and dermoscopic examinations. Thus, histopathological examination is essential to confirm the diagnosis [1].

Clinical diagnosis of poromas is often inaccurate, especially when it occurs in an uncommon location. It is easily mistaken for pyogenic granuloma, chronic ulcer, basal cell carcinoma, amelanotic melanoma, or seborrheic keratosis [10]. Pedunculated, nontender, rubbery to firm skin growths with smooth surfaces, as in our case, over the lower limb raised the suspicion of a fibroepithelial polyp (skin tag) as the first possibility with other differentials including pyogenic granuloma, hemangioma, neuroma, neurilemmoma, and less commonly, benign or malignant adnexal tumors [8,10]. After consulting with the patient and various physicians, we surgically excised the lesion and sent it for histopathology examination.

The histopathology of poromas is different from the skin tag. It shows broad anastomosing bands come from the epidermis and extend into the dermis and consist of uniform, mostly cuboidal cells with basophilic nuclei with occasional vacuolated areas (due to glycogen). Cystic spaces and ductal lumina may also be present. This is to be seriously looked for any malignant changes and differentiated from porocarcinoma, which shows mainly cytological atypia and infiltrative growth. However, irritated poromas may also show focal atypia, mitosis, or even occasional necrosis [2]. Up to 0.01% of poromas can progress to malignancy (porocarcinoma), highlighting the special need for timely diagnosis and proper treatment [2,8].

Histopathology is a relatively straightforward diagnostic tool for such unusual skin tumors, so clinicians should consider the clinicopathological correlation to confirm the diagnosis and exclude malignant transformation. Treatment of poroma usually consists of simple excision. However, if the lesion recurs, bleeding occurs, presents with ulceration, or accelerates growth, porocarcinoma should be excluded and properly assessed. Some authors suggested surgical margins of 3-10 mm that are supposed to decrease the recurrence rate, but there is still no evidence-based consensus on a recommended excision margin [1].

## Conclusions

Aside from being a rare surgical or dermatological entity, mentions of nonacral poromas are even sparser in the literature, and very few cases have been reported on the thigh. The definitive treatment of poromas is the complete surgical excision with clear margins because local recurrence is possible. It is, therefore, important to consider histopathology examination for all excised skin tags and to have a basic knowledge of this uncommon process, despite its benign nature.
